# Skeletal muscle and performance adaptations to high-intensity training in elite male soccer players: speed endurance runs versus small-sided game training

**DOI:** 10.1007/s00421-017-3751-5

**Published:** 2017-11-08

**Authors:** Dan Fransson, Tobias Schmidt Nielsen, Karl Olsson, Tobias Christensson, Paul S. Bradley, Ioannis G. Fatouros, Peter Krustrup, Nikolai Baastrup Nordsborg, Magni Mohr

**Affiliations:** 1 Department of Food and Nutrition, and Sport Science, Center for Health and Human Performance, University of Gothenburg, Gothenburg, Sweden; 2 Department of Nutrition, Exercise and Sports, University of Copenhagen, Copenhagen, Denmark; 3 Department of Health and Caring Sciences, Linnaeus University, Kalmar, Sweden; 4 Research Institute of Sport and Exercise Sciences, Liverpool John Moores University, Liverpool, UK; 5 School of Physical Education and Sport Science, University of Thessaly, Karies, Trikala, Greece; 6 Department of Sports Science and Clinical Biomechanics, SDU Sport and Health Sciences Cluster (SHSC), University of Southern Denmark, Odense, Denmark; 7 Sport and Health Sciences, College of Life and Environmental Sciences, University of Exeter, Exeter, UK; 8 Faculty of Health Sciences, Centre of Health Science, University of the Faroe Islands, Jónas Broncks gøta 25. 3rd floor, Tórshavn, Faroe Islands

**Keywords:** Antioxidant capacity, Intermittent exercise, Muscle fatigue, Muscle oxidative capacity, Na^+^–K^+^ ATPase activity, Football

## Abstract

**Purpose:**

To examine the skeletal muscle and performance responses across two different exercise training modalities which are highly applied in soccer training.

**Methods:**

Using an RCT design, 39 well-trained male soccer players were randomized into either a speed endurance training (SET; *n* = 21) or a small-sided game group (SSG; *n* = 18). Over 4 weeks, thrice weekly, SET performed 6–10 × 30-s all-out runs with 3-min recovery, while SSG completed 2 × 7–9-min small-sided games with 2-min recovery. Muscle biopsies were obtained from m. vastus lateralis pre and post intervention and were subsequently analysed for metabolic enzyme activity and muscle protein expression. Moreover, the Yo–Yo Intermittent Recovery level 2 test (Yo–Yo IR2) was performed.

**Results:**

Muscle CS maximal activity increased (*P* < 0.05) by 18% in SET only, demonstrating larger (*P* < 0.05) improvement than SSG, while HAD activity increased (*P* < 0.05) by 24% in both groups. Na^+^–K^+^ ATPase α_1_ subunit protein expression increased (*P* < 0.05) in SET and SSG (19 and 37%, respectively), while MCT4 protein expression rose (*P* < 0.05) by 30 and 61% in SET and SSG, respectively. SOD2 protein expression increased (*P* < 0.05) by 28 and 37% in SET and SSG, respectively, while GLUT-4 protein expression increased (*P* < 0.05) by 40% in SSG only. Finally, SET displayed 39% greater improvement (*P* < 0.05) in Yo–Yo IR2 performance than SSG.

**Conclusion:**

Speed endurance training improved muscle oxidative capacity and exercise performance more pronouncedly than small-sided game training, but comparable responses were in muscle ion transporters and antioxidative capacity in well-trained male soccer players.

## Introduction

Human skeletal muscle has a high plasticity and adapts to various exercise modalities due to specific training-induced stimuli and the type and magnitude of these adaptations impact on high-intensity exercise (Nader and Esser 2001). For example, 2–6 weeks of high-intensity intermittent training termed ‘speed endurance training’ increased mitochondrial protein content and maximal activity of key enzymes (Burgomaster et al. 2008; Gibala et al. 2006), whereas mitochondrial enzyme activity remained constant after 6 weeks of continuous training at a lower exercise intensity (Cochran et al. 2014). Speed endurance training for periods of 2–9 weeks has been extensively studied (Bangsbo et al. 2009; Christensen et al. 2011; Gunnarsson et al. 2013; Laursen and Jenkins 2002; Mohr et al. 2007; Thomassen et al. 2010) and has been shown to improve high-intensity intermittent exercise performance (Gibala et al. 2012; Iaia and Bangsbo 2010). Improved muscle function may possibly be caused by an increased expression of muscle ion transporters, such as the Na^+^–K^+^ ATPase, monocarboxylate transporters and the Na^+^/H^+^ exchanger, which may facilitate ion handling capacity. Moreover, speed endurance training has been shown to elicit larger performance gains than sprint training (15 × 6 s sprints) (Mohr et al. 2007) and endurance training (prolonged submaximal runs) (Iaia et al. 2008). Moreover, muscle ion transport capacity and high-intensity exercise performance are improved with the inclusion of speed endurance training, even when the training volume is markedly reduced (Iaia et al. 2008; Thomassen et al. 2010), or when speed endurance training is added to normal training (Gunnarsson et al. 2013). In the past studies, speed endurance training protocols have been included at the expense of less intensive training. However, it is currently unknown if additional speed endurance training elicits a different muscle and performance response compared to additional training at moderate intensity in high-intensity athletes such as competitive soccer players. In competitive soccer, different training modalities are applied (Ade et al. 2014), but a major part of the fitness training is conducted as small-sided games (Dellall et al. 2011). However, limited information exists on the efficiency of this training modality compared to more controlled running drills. In a study by Ade et al. (2014), the characteristics of speed endurance running and small-sided game where compared and marked differences were observed. However, no study has yet tested the muscle and performance adaptations of speed endurance versus small-sided game training.

In a high-intensity intermittent sport such as soccer, the physical demands are complex, encompassing both high endurance capacity and fatigue resistance during high-intensity exercise (Fransson et al. 2017; Mohr et al. 2005). Endurance performance in a soccer game is related to muscle oxidative capacity, while muscle Na^+^-K^+^ ATPase protein expression displays a strong correlation to high-intensity exercise performance in a game (Mohr et al. 2016b). As a consequence of these observations, studies relating adaptations in both endurance and high-intensity exercise performance to muscular adaptations are highly warranted.

During soccer exercise the oxidative stress is highly upregulated (Mohr et al. 2016a) challenging antioxidative enzymes such as superoxide dismutase (SOD) and catalase (CAT) that prevent oxidative damage (Jackson et al. 2011). Exercise training may increase the skeletal muscle antioxidative capacity, as evidenced by upregulated activity in GPX, SOD1 and SOD2 in untrained populations (Gliemann et al. 2014). However, limited studies have addressed whether the muscular antioxidant capacity is augmented by speed endurance training in an athletic population. High-intensity interval training may upregulate the muscle antioxidative capacity and lower systemic oxidative stress (Atalay et al. 1996), even after just a very brief training exposure (e.g. 3 weeks) (Bogdanis et al. 2013; Scribbans et al. 2014). When high-intensity exercise protocols were compared to continuous endurance training of lower intensity, antioxidant enzymes such as SOD, GPX, and catalase (CAT) seemed to be upregulated to a greater (Songstad et al. 2015; Tucker et al. 2015) or to the same (Lu et al. 2015) extent in the former than in the latter. However, these studies did not include trained athletes, who are likely to have a high muscle antioxidant capacity, and may require additional high-intensity training to induce further redox-dependent adaptations such as mitochondrial biogenesis.

Thus, this study aimed to examine muscle oxidative capacity, ion transporters, and antioxidant adaptations, as well as performance responses to 4 weeks of additional speed endurance training or small-sided game training in trained male soccer players. It was hypothesized that speed endurance training would result in more pronounced adaptations in (1) high-intensity intermittent performance, (2) skeletal muscle oxidative capacity, and (3) muscle ion transporters and antioxidant proteins compared to small-sided game training at moderate exercise intensity.

## Methods

### Subjects

Thirty-nine trained semi-professional male soccer players (mean ± SD, age 21.1 ± 2.4 years; height 184 ± 7 cm; body mass 77.5 ± 7.8 kg) representing two teams in the third division in Sweden volunteered to participate in the study. The participants represented all outfield positions (central defenders *n* = 7, full-backs *n* = 8, central midfielders *n* = 6, wide midfielders *n* = 10, attackers *n* = 8). The players trained four times a week and did not participate in match play during the intervention period. The intervention was initiated 2 weeks into the pre-season in January 2016. All participants had played competitive soccer for at least 5 years prior to the start of the study. Players who had been injured within a period of 6 weeks prior to the start of the study were excluded from the study. All participants were informed of potential risks and discomforts associated with the experiment before giving their written consent to participate according to the guidelines of the Helsinki Declaration. The study was approved by the local ethics committee in Gothenburg (Dnr: 687-15).

### Experimental design

The study applies a randomized controlled design. The participants were randomized to a speed endurance training group (SET; *n* = 21) or a small-sided game training group (SSG; *n* = 18). Players from the two teams were randomized within teams and playing position to ensure and equal representation of both intervention groups. The two different types of training were added to the players’ normal training programs three times a week for 4 weeks in total. We have chosen to compare SET and SSG, since pilot studies have demonstrated a markedly higher exercise intensity during SET compared to SSG, which also is supported by Mohr and Krustrup (2016). Pre and post intervention physical performance tests were performed to measure the impact of the two intervention training protocols on high-intensity exercise performance. Moreover, a muscle biopsy was obtained pre and post intervention from the *m*. vastus lateralis of the dominant leg. The muscle tissue was analysed for metabolic enzyme maximal activity and expression of a wide range of skeletal muscle proteins. In addition, activity pattern during training was assessed and capillary blood samples were taken during training and analysed for blood lactate concentration.

### Experimental protocol

SET (*n* = 21) performed speed endurance production training (Mohr and Krustrup 2016), three times a week for a 4-week period. The training comprised a 20-m straight forward run and 90° turn followed by a 10-m forward run and 180° turn, then another 20-m forward run, another 180° turn, and finally a 40-m forward run. This specific drill was chosen after pilot testing with GPS technology and with feedback from the coaches. Participants in SET were informed to run with maximum effort during the entire drill and were continuously verbally encouraged. The drill was performed individually as a time trial drill in 30-s intervals separated by 150 s of passive recovery. The course of the drill ensured that players could perform the drill several times without stopping to ensure that the drill lasted exactly 30-s. The number of exercise intervals was six during the first intervention week, eight during the second and third weeks, and ten during the fourth week. The SSG group performed a 6-a-side soccer game with goal keepers on a pitch 40 m long and 32 m wide three times a week for 4 weeks (Dellal et al. 2011). Pilot studies showed that this specific drill had a markedly lower exercise intensity compared to SET. The training was performed in two intervals lasting 7 min in the first week, two intervals lasting 8 min in the second and third weeks, and two intervals of 9 min in the fourth week. The participants had a passive recovery interval of 2 min between exercise intervals. The 6-a-side games were played with normal rules and players were verbally encouraged. The two different drills were performed at the end of the normal training (~ 60 min) three times a week. The normal training included a ~ 15-min warm-up, ~ 15 min of technical training, and ~ 30 min of tactical training. The players were familiarized with the drills prior to the study. The intervention took place during the pre-season and was conducted outdoors on an artificial pitch. The environmental temperature during the intervention period was 3–8 °C. Forty-five players started the study. There were six drop-outs due to injury (*n* = 4) or illness (*n* = 2). These players were excluded from the measures and analysis, giving a final sample of 39. During the study, participants were instructed to follow their usual diet before all testing and training sessions.

### Muscle analyses

Twelve players did not give informed consent to have muscle biopsies taken but underwent all other measurements. Thus, 27 participants had a muscle biopsy taken (~ 70–120 mg wet weight) in resting conditions a week before the start of the intervention. The biopsies were obtained with the subjects lying in the supine position on a portable bed. This procedure was repeated in the week after the intervention 3 days after the last training session. The muscle tissue was immediately frozen in liquid nitrogen and stored at − 80 °C. The frozen sample was weighed after freeze-drying as well as 1 h later to correct for the water content. After freeze-drying, the muscle samples were dissected free of blood, fat, and connective tissue. Next, 1–2 mg dry weight muscle tissue was extracted in 1 M HCl, hydrolyzed at 100 °C for 3 h, and the glycogen content determined using the hexokinase method. Maximal citrate synthase (CS), 3-hydroxyacyl-CoA-dehydrogenase (HAD), and phosphofructokinase (PFK) activities were determined fluorometrically in triplicate for each biopsy on a separate piece of muscle from the biopsy, as described by Lowry (Lowry and Passonneau 1972), and these analyses displayed CV values between 4 and 7%.

### Western blotting

The protein expression was determined as described by Thomassen et al. (Thomassen et al. 2010). In short, ~ 2 mg of the dissected human muscle tissue was homogenized in duplicate for each biopsy (Qiagen Tissuelyser II, Retsch GmbH, Haan, Germany) in a fresh batch of buffer containing (in mM): 10% glycerol, 20 Na-pyrophosphate, 150 NaCl, 50 HEPES (pH 7.5), 1% NP-40, 20 β-glycerophosphate, 2 Na3VO4, 10 NaF, 2 PMSF, 1 EDTA (pH 8), 1 EGTA (pH 8), 10 µg/ml aprotinin, 10 µg/ml leupeptin, and 3 benzamidine. Afterwards, the samples were rotated end over end for 1 h at 4 °C, centrifuged at 18,320 *g* for 20 min at 4 °C, and the supernatant (lysate) used for further analysis. Total protein concentration in each sample was determined by a bovine serum albumin (BSA) standard kit (Pierce), and samples were mixed with 6 × Laemmli buffer (7 ml 0.5 M Tris-base, 3 ml glycerol, 0.93 g DTT, 1 g SDS, and 1.2 mg bromophenol blue) and ddH_2_0 to obtain similar concentrations in all samples.

An equal amount of total protein was loaded in each well of pre-casted gels (Bio-Rad Laboratories, USA) and all samples from each subject were loaded side by side on the same gel. Proteins were separated according to their molecular weight by sodium dodecyl sulphate polyacrylamide gel electrophoresis (SDS–PAGE) and then transferred semi-dry to polyvinylidene difluoride (PVDF) membranes (Bio-Rad). The membranes were blocked in either 2% skimmed milk or 3% BSA in Tris-buffered saline including 0.1% Tween-20 (TBST) before overnight incubation in primary antibody at 4 °C. Subsequently, the membranes were washed for 2 × 10 min in TBST and incubated for 1 h at room temperature in horseradish peroxidase (HRP)-conjugated secondary antibody. The membranes were then washed for 3 × 15 min in TBST before the bands were visualized with ECL (Millipore) and recorded with a digital camera (ChemiDoc MP Imaging System, Bio-Rad Laboratories, USA). Quantification of the Western blot band intensity was carried out using Image Lab version 4.0 (Bio-Rad Laboratories, USA), and the mean of samples A and B in arbitrary units was used as the final result estimating the protein expression in each subject. To evaluate intervention-induced changes in protein expression, post versus pre, band signal intensity ratios were calculated for each individual and values ≥ 3 excluded from further analysis to avoid statistical type II errors.

Prior to the final analyses, the primary antibodies were optimized using human mixed muscle standard lysates to ensure that the band signal intensity was located on the linear part of the specific antibody standard curve. The primary antibodies used to detect the expression of the investigated proteins were diluted in either 2% skimmed milk [polyclonal Na^+^–K^+^ ATPase α_2_ (07-674, Millipore), monoclonal β_1_ (MA3-930, Thermo Scientific), polyclonal FXYD1 (13721-1-AP, Datasheet), polyclonal MCT4 (AB3316P, Millipore), polyclonal SOD2 (06-984, Millipore), polyclonal CAT (ab1877, Abcam), polyclonal GLUT4 (PA1-1065, Thermo Fisher Scientific) and polyclonal GS (3893, Cell Signaling Technology)] or 3% BSA [monoclonal Na^+^-K^+^ ATPase α_1_ (alfa6F, Developmental Study Hydridoma Bank, University of Iowa, USA), polyclonal SOD1 (574597, Millipore) and monoclonal NHE1 (MAB3140, Chemicon)] in TBST. Antibodies targeting SOD1 and SOD2 were kindly provided by Prof. H. Pilegaard, University of Copenhagen. Applied secondary antibodies were HRP-conjugated goat anti-rabbit (4010-05, Southern Biotech), rabbit anti-sheep (P-0163, DAKO) and goat anti-mouse (P-0447, DAKO). The muscle buffer capacity was measured after having adjusted the pH of the sample to 7.1 with 0.01 M NaOH. The sample was titrated to pH 6.0 by serial additions of 0.01 M HCl, followed by titration back to pH 7.1 by serial additions of 0.01 M NaOH. The pH was assessed after each addition. The non-HCO_3_ physiochemical buffer capacity was determined from the number of moles of H^+^ required to change the pH from 7.1 to 6.5 and was expressed as millimoles H^+^ per kilogram dry weight per unit of pH.

### Physical and physiological training response

During a representative intervention training session in week 3, blood samples were obtained from the fingertip for blood lactate analysis according to standard procedure (Pettersen et al. 2014) to have an indication of the glycolytic loading in the two training interventions. The baseline blood was taken 5 min before normal training. In SET, blood was then taken after intervals 4 and 8 analogous to previous studies (Ade et al. 2014), while in the SSG group blood was obtained after the first and last intervals. Capillary blood was frozen and stored at − 80 °C until analysed for lactate using a Biosen analyzer (Biosen C-line, EKF-diagnostic GmbH, Magdeburg, Germany; see Pettersen et al. 2014).

Activity pattern characteristics during the additional training were quantified using a 10-Hz S5 global positioning system (GPS) (Catapult Innovations, Melbourne, Australia) on three occasions per player (in weeks 1, 2 and 3). This was done to compare the external physical loading of the two training interventions (see also Mohr and Krustrup 2016). The GPS units were placed between the shoulder blades in a harness tight to the body to restrict movement artifact. The GPS has previously been shown to provide a valid and reliable measure of instantaneous velocity during acceleration, deceleration, and constant motion (Varley et al. 2012). Time motion characteristics were quantified as total distance (TD), high-intensity running distance (HIR), and high-speed running distance (HSR), and set at > 0 km/h, > 14 km/h, and > 21 km/h, respectively (see Fransson et al. 2017). Intense accelerations (Ia) and intense decelerations (Id) were also analysed and set at > 3 m/s^2^.

### Physical performance tests

Pre and post intervention, the participants performed a repeated sprint test (RST) comprising 5 × 30-m straight-line sprints separated by 25 s of active recovery (easy jogging back to the start line) (Nybo et al. 2013). The test has been shown to be reliable (Castagna et al. 2017) and to possess a high reproducibility (CV < 1%; Bangsbo and Mohr 2012). RST performance was determined as mean sprinting time, which has been shown to be reliable (Spencer et al. 2006). Also a fatigue index was calculated as the percentage decline in sprint performance from the first to the last sprint. The test was chosen as a measure of repeated sprint ability in relation to soccer (Bangsbo and Mohr 2012). The participants also performed an arrowhead agility test (AAT) consisting of four trials, two right and two left, according to previous studies (Di Mascio et al. 2015; Noon et al. 2015). This test was applied as a measure of soccer-specific agility and has a high reproducibility (CV < 1% Bangsbo and Mohr 2012; Di Mascio et al. 2015). Cones are placed in an arrowhead shape, with one set of cones indicating the start and finish line. The RST and AAT were performed in an indoor hall with a temperature of ~ 20 °C. The sprint times were measured by photocell gates placed 1.0 m above ground using Muscle Lab V8 (Bosco System, Rome, Italy) photocells with a precision of 0.001 s. Each sprint was initiated from a standing position with the arms raised to chest height 50 cm behind the photocell gate, which started a digital timer. The time of each sprint was recorded and total sprint time calculated. A fatigue index was also calculated between the fastest time and the slowest sprint in the RST as well as the accumulated total time of the five sprints. The participants were familiarized with the tests in two pre-trials prior to commencement of the study. Finally, pre and post intervention with 3 days of recovery after the RST and AH, a Yo–Yo intermittent recovery test level 2 (Yo–Yo IR2) was performed following a 10-min standardized warm-up (see Bangsbo and Mohr 2012). The Yo–Yo IR2 test has a high sensitivity and a relatively high reproducibility (CV = 9.6%, Krustrup et al. 2006) and is correlated to high-intensity running in a soccer game as well as muscle variables of importance for fatigue resistance during intense exercise (Mohr et al. 2016b). The test was performed outside on artificial grass at an environmental temperature of ~ 4–8 °C. The test consists of repeated 2 × 20-m runs back and forth between a start and finish line at progressively increasing speeds until exhaustion, controlled by audio bleeps. Between each running bout, the participants have a 10-s active recovery period in which they have to jog around a cone placed 5 m behind the starting line. The participants run until exhaustion, as previously described (Bangsbo and Mohr 2012). Yo–Yo IR2 testing was part of the clubs’ test battery, so the participants were familiar with the test procedures.

### Statistical analyses

Data are presented as means ± SD. Differences in change score in physical test performance and muscle responses between SET and SSG, as well as within-group differences, were evaluated using a two-way ANOVA test. Activity profiles and training responses were compared using a two-way ANOVA with repeated measurements. When a significant interaction was detected, data were subsequently analysed using a Newman–Keuls post hoc test. 95% confidence intervals are presented and effect size (ES; Cohens d calculation) was used to assess the magnitude of the differences and considered trivial (< 0.2), small (0.2–0.6), moderate (0.6–1.2), large (1.2–2.0) and extremely large (> 2.0) as suggested by Hopkins et al. (2009). A significance level of 0.05 was chosen.

## Results

### Exercise training

The SSG group (*n* = 13) covered 24% greater (*P* < 0.05) distance in total during the assessed intervention trainings compared to SET (*n* = 17; Table 1). However, SET covered approximately four- and 16-fold more (*P* < 0.05) high-intensity and high-speed running, respectively, than SSG (Table 1). In addition, the SET intervention induced 41 and 163% more intense accelerations and decelerations in comparison to SSG based on the assessed sessions (Table 1). Finally, capillary blood lactate concentrations during SET (*n* = 16) were more than twice as high in comparison to SSG (*n* = 12; Table 1).

**Table 1:** Activity pattern and blood lactate during training

Group	TDC (m)	HIR (m)	HSR (m)	Ia (*n*)	Id (*n*)
SET (*n* = 17)	1364 ± 84*	826 ± 102*	239 ± 53*	38 ± 9*	50 ± 8*
SSG (*n* = 13)	1683 ± 348	180 ± 133	14 ± 15	27 ± 14	19 ± 11

Group	Baseline	Post 4-min	Post 8-min		
Blood lactate concentration (mmol·L^−1^)					
SET (*n* = 16)	3.4 ± 1.7	11.8 ± 2.8*	13.7 ± 3.4*		
SSG (*n* = 12)	2.6 ± 1.9	4.7 ± 2.0	4.8 ± 2.3		

*TDC* Total distance covered, *HIR* high-intensity running, *HSR* high speed running, *Ia* intense accelerations, *Id* decelerations (upper panel) and blood lactate concentrations (lower panel) during training in SET and SSG

*Significant different from SSG. Significant level *P* < 0.05. Data are means ± SD

### Muscular metabolic enzyme expression and substrate level

**Fig. 1:**
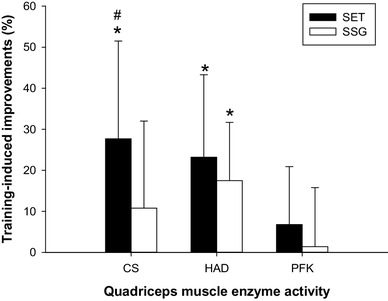
Relative change from pre to post intervention in citrate synthase (CS), 3-hydroxyacyl-CoA-dehydrogenase (HAD), and phosphofructokinase (PFK) maximal enzyme activity determined in muscle tissue from vastus lateralis muscle in SET (*n* = 15; solid bars) and SSG (*n* = 11; open bars). ^#^Significant between-group differences in change score. *-Significant within-group difference from pre to post intervention. Significance level *P* < 0.05. Data are means ± SD

**Fig. 2:**
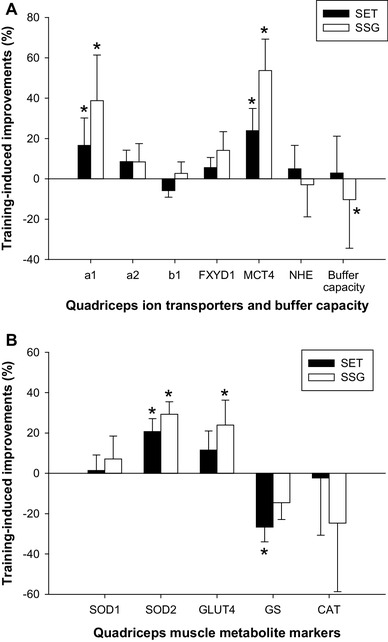
Relative change from pre to post intervention in Na^+^-K^+^ ATPase α_1,_ α_2,_ β_1 and_ FXYD1, MCT4 and NHE1 protein expression, as well as buffer capacity (**a**), and SOD1, SOD2, GLUT4, GS and CAT protein expression (**b**) determined in muscle tissue from vastus lateralis muscle in SET (*n* = 15; solid bars) and SSG (*n* = 12; open bars). ^#^Significant between-group differences in change score. *Significant within-group difference from pre to post intervention. Significance level *P* < 0.05. Data are means ± SD

Skeletal muscle CS maximal activity increased (ES = 1.6, CI = 9.2–3.1, *P* < 0.05) over the intervention period from 25.5 ± 3.1 to 30.0 ± 3.1 µmol·g^−1^·min^−1^ in SET (*n* = 15) only, with a larger (*P* < 0.05) change score compared to SSG (*n* = 11; Fig. 1). Muscle HAD maximal activity was also elevated (ES = 1.1, CI = 5.1–1.3, *P* < 0.05) post intervention in SET (15.3 ± 1.9 to 18.5 ± 4.0 µmol·g^−1^·min^−1^; *n* = 15) and in SSG (ES = 1.3, CI = 6.4–1.4) (15.7 ± 2.8 to 19.5 ± 3.0 µmol·g^−1^·min^−1^; *n* = 11) with no between-groups difference (Fig. 1). Muscle PFK maximal activity showed no changes between pre and post analysis and no between-groups difference (Fig. 1). GLUT-4 protein expression responded in SSG (ES = 0.6, CI = 5–75%) (40 ± 54%; *n* = 12; *P* < 0.05), with no changes in SET (Fig. 2b), while GS protein expression showed a negative change score (*P* < 0.01) of − 22 ± 30% (*n* = 15) in SET (ES = 0.2, CI = − 38 to − 5%) with no change in SSG (Fig. 2b). Resting muscle glycogen concentration was 679 ± 91 and 678 ± 85 mmol·kg^−1^ d.w. in SSG (in front of (*n* = 12)), pre intervention, but was elevated (*P* < 0.05) to 758 ± 143 and 853 ± 189 mmol·kg^−1^ d.w. post intervention. Resting muscle glycogen concentration tended (*P* = 0.09) to increase more in SSG than in SET.

### Muscular ion-regulatory enzyme expression

Protein expression for the α_1_ Na^+^–K^+^ ATPase subunit increased in SET (ES = 0.5, CI = 46–34%) (19 ± 26%; *n* = 15; *P* < 0.05) and SSG (ES = 0.7, CI = 13–63%) (37 ± 41%; *n* = 12; *P* < 0.01), with a tendency (*P* = 0.07) for a greater change score in SSG (Fig. 2a). In contrast, no between-group or within-group differences were detected in the α_2_, β_1_ and FXYD1 Na^+^–K^+^ ATPase subunits post intervention (Fig. 2a). MCT4 protein expression was upregulated in response to both interventions (SET: 30 ± 41%; *n* = 15; ES = 0.3, CI = 7–52%, *P* < 0.05; SSG: 61 ± 49%; *n* = 12; ES = 0.7, CI = 30–92%, *P* < 0.01), with no between-group effects (Fig. 2a). No between-group or within-group effects were observed in NHE1 protein expression or muscle buffer capacity (Fig. 2a).

### Muscular antioxidative enzyme expression

SOD2 protein expression increased in SET (ES = 0.8, CI = 10–46%) and SSG (ES = 1.1, CI = 19–55%) [28 ± 32% (*n* = 15) and 37 ± 29% (*n* = 12), respectively; *P* < 0.05], while no intervention-induced changes occurred in protein expression of SOD1 (Fig. 2b). The SET and SSG interventions did not affect CAT protein expression (Fig. 2b).

### Exercise performance

**Fig. 3:**
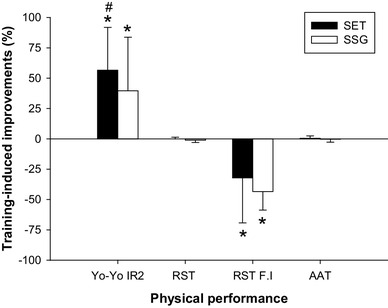
Relative change from pre to post intervention in Yo–Yo intermittent recovery test, level 2 (Yo–Yo IR2), repeated sprint test (RST), and RST fatigue index (RST_FI_) and arrowhead agility test (AAT) performance in SET (*n* = 21; solid bars) and SSG (*n* = 18; open bars). ^#^Significant between-group differences in change score. *Significant within-group difference from pre to post intervention. Significance level *P* < 0.05. Data are means ± SD

At baseline Yo–Yo IR2 performance was 569 ± 147 and 563 ± 145 m in SET and SSG, respectively. Yo–Yo IR2 performance increased in SET (ES = 1.9, CI = − 386.8 to − 357.9, *P* < 0.05) (*n* = 21) and SSG (ES = 1.3, CI = − 387.1 to 158.0, *P* < 0.05) (*n* = 18) by 323 ± 125 and 222 ± 113 m, respectively, with a 39% greater change score (*P* < 0.05) in SET than in SSG (Fig. 3). RST performance was unaltered in both SET and SSG, as mean sprint time did not change during the intervention. However, the fatigue index improved (*P* < 0.001) in both groups (Fig. 3). AAT performance did not change during the intervention period (Fig. 3).

## Discussion

The principal findings of the present study were that 4 weeks of additional speed endurance training induced more pronounced improvement of shuttle-run performance and CS maximal activity, whereas small-sided games tended to induce more pronounced increases in the expression of Na^+^–K^+^ ATPase α_1_ and GLUT4 as well as a higher level of muscle glycogen. The observed increases of HAD maximal activity, as well as MCT4 and SOD2 expression were comparable between groups. Thus, the training outcome is clearly different between the applied protocols in terms of exercise capacity, as well as muscle phenotype.

In the present study, SET performed as all-out 30-s runs induced superior effects on skeletal muscle oxidative capacity compared to SSG training in well-trained soccer players. SSG are highly applied in competitive soccer training compared to SET (Dellal et al. 2011), but the two methods have not been compared in relation to performance and physiological impact. We demonstrate that CS maximal activity in the quadriceps muscle showed greater responses in SET than in SSG (see Fig. 1). In contrast, muscle HAD maximal activity was enhanced to a similar extent after both training protocols. Thus, these findings indicate that exercise training-induced adaptations in skeletal muscle oxidative capacity are not strongly associated with metabolic flow during training. For example, the SSG approach has been shown to have a marked aerobic component (Halouani et al. 2014) which is also supported by a ~ 25% longer running distance in SSG than SET (see Table 1). In divergence, SET demonstrated a markedly higher exercise intensity as well as glycolytic activity, as observed in previous studies (Iaia et al. 2009; Mohr et al. 2007). Despite this, and in contrast to the oxidative markers, muscle PFK activity was not upregulated in response to the intense training regime, which is in line with other speed endurance training studies (Harmer et al. 2000; Mohr et al. 2007; Nordsborg et al. 2015), supporting the notion above. The finding of upregulated mitochondrial capacity after SET has been reported in several other studies in both untrained individuals (Burgomaster et al. 2008; Gibala et al. 2006; Nordsborg et al. 2015) and trained athletes (Iaia and Bangsbo 2010; Skovgaard et al. 2016). SET appears to increase muscle oxidative capacity when added to normal training (Gunnarsson et al. 2013), as in the present study, and when performed in conjunction with lowered training volume (Christensen et al. 2011; Nordsborg et al. 2015). Recently, MacInnis et al. (MacInnis et al. 2017) also demonstrated that single-leg cycling performed in an interval compared to a continuous manner elicited superior skeletal muscle mitochondrial adaptations despite equal total work. Collectively, this research supports the present study that SET upregulates oxidative capacity in skeletal muscle and appear to be superior to endurance-based training even in well-trained athletes. A potential mechanism may be that high-intensity interval training enhances aerobic metabolism (Harmer et al. 2000) via upregulation of PGC-1α mRNA expression (Nordsborg et al. 2010; Skovgaard et al. 2016) and subsequently improved mitochondrial biogenesis.

An interesting finding in the current study was a nearly 40% greater improvement in Yo–Yo IR2 test performance in the SET compared to the SSG training group. In contrast, RST and agility performance was unaltered, but the fatigue index in the RST displayed comparable improvement in both SET and SSG. It should be noted that the application of fatigue indexes in RST is questionable (Oliver 2009). There is strong backing in the literature for the proposition that SET increases high-intensity exercise performance in both habitually active (Mohr et al. 2007) and trained populations (Iaia et al. 2009; Mohr and Krustrup 2016; Thomassen et al. 2010). For example, it was demonstrated by Thomassen et al. (Thomassen et al. 2010) that repeated sprint ability improved after only 2 weeks of SET even after the training volume was markedly reduced in well-trained soccer players. Fatigue during high-intensity exercise has been linked to depolarization of the resting muscle membrane potential caused by disturbance in the muscle ion homeostasis, with a large K^+^ efflux from the muscle cell and accumulation in the muscle interstitial fluid being suggested to play a central role (McKenna et al. 2008). Thus, an upregulation in muscle Na^+^–K^+^ ATPase protein expression, may be beneficial for delaying the onset of fatigue during intense exercise. However, in the present study, SET and SSG training groups improved (19 and 37%, respectively) the protein expression of the α_1_ Na^+^–K^+^ ATPase subunit, with a tendency (*P* = 0.07) for more marked improvement in the SSG group (Fig. 2a), demonstrating that the more pronounced improvements in Yo–Yo IR2 performance in SET are unrelated to the altered α_1_ Na^+^–K^+^ ATPase expression.

Muscle MCT4 protein expression increased in both training groups with no between-group differences, while no changes occurred in NHE1 protein expression (Fig. 2a). Thus, the muscle lactate^−^ and H^+^ regulation capacity were partly improved by both interventions, despite a decline in muscle buffer capacity after the SSG intervention (Fig. 2a). The decline in buffer capacity may have influenced the inferior exercise performance adaptations in SSG in comparison to SET. Blood lactate concentration during training was markedly higher in SET than in SSG (see Table 1), indicating a higher muscle lactate production and a potentially greater degree of muscle acidification in SET (Mohr et al. 2007), which potentially have maintained muscle buffer capacity in SET. In contrast, the adaptive response to exercise training in MCT4 and NHE1 does not appear to be highly influenced by the flow rate through these transporters.

Muscle antioxidant capacity was upregulated in both training groups, with SOD2 muscle protein expression elevated by 28 and 37% in SET and SSG, respectively (Fig. 2b). SOD quenches the superoxide anion (Jiang et al. 2014), whereas antioxidant enzymes seem to inhibit the translocation of NF-κΒ to the nucleus, thereby contributing to the attenuation of exercise-induced oxidative stress and inflammatory cascades induced by intense exercise (Azevedo-Martins et al. 2003). This is particularly important for high-intensity intermittent exercise, which induces a marked rise in oxidative stress and inflammatory responses (Mohr et al. 2016a). Conventional endurance training may increase the expression and activity of antioxidant enzymes such as GPX, SOD, and CAT in skeletal muscle following several weeks of exposure (Evelo et al. 1992; Fatouros et al. 2004; Oh-ishi et al. 1997). However, similar data from human muscle tissue following high-intensity training regimes such as speed endurance regimes are sparse. One study found that only three speed training sessions induced an elevation in antioxidant status (Shing et al. 2007), which coincides with the elevation in SOD2 protein expression after only 4 weeks of high-intensity intermittent training in our study. The supportive findings on SOD2 following speed endurance training have been reported in two animal studies (Lu et al. 2015; Tucker et al. 2015), and one human study with recreationally active participants (Scribbans et al. 2014). Repeated exposure to intense exercise-induced ROS generation and inflammation is suggested to be a prerequisite for SOD upregulation (Powers et al. 1993), which may indicate that training intensity is an important variable for the adaptive response. However, statistically the two training protocols in the present study were equally effective in upregulating SOD2. The elevated expression in SOD2 proteins has been linked to the muscle oxygen consumption during training (Jenkins et al. 1984), since a close relationship has previously been reported for antioxidant enzymes and adaptation in TCA cycle enzymes (Burgomaster et al. 2006, 2007; Gibala et al. 2012; Laughlin et al. 1990). In the present study, the cytosol-based SOD1 protein remained unaffected by the training intervention, whereas SOD2 located in the mitochondrial intermembrane space increased and may be indicative of interplay between antioxidant reserves and mitochondrial adaptations to training. Our data partly verify this notion, with the SOD2 expression rise which coinciding with the upregulation of muscle oxidative capacity, as evidenced by the rise in CS activity in the SET group. However, no intervention-induced adaptations were detected in SSG despite the ~ 40% elevation in SOD2 expression, which speaks in favour of other muscle signalling mechanisms for mitochondrial biogenesis. In addition, no correlation was found between the delta change in CS and SOD2 during the training intervention period (data not shown). Rats bred to have high running capacity were characterized by having high muscle SOD2 activity, suggesting that increased endurance is characterized by an increased molecular network of resistance to oxidative stress (Tweedie et al. 2011). However, in the present study, no relationship was seen between exercise performance adaptation and upregulation of SOD2. Further studies are warranted to elucidate redox adaptations to exercise training in trained human skeletal muscle.

Resting muscle glycogen increased in both groups after the training intervention, which is a common finding in other training studies (Nordsborg et al. 2015; Randers et al. 2010). However, muscle glycogen tended to increase more in SSG compared to SET. This is supported by the fact that muscle glucose transport capacity, marked by GLUT4 protein expression, was enhanced by 40% in SSG with no change after SET (Fig. 2b), while glycogen synthase protein expression was down regulated in SET. Thus, the SSG intervention was apparently more efficient at improving muscle glycogen storage capacity, despite that no difference was detected in resting glycogen measured in muscle homogenate. In contrast to the present study, a number of studies have found increased muscle GLUT4 content (Little et al. 2010), as well as resting muscle glycogen (Nordsborg et al. 2015), after high-intensity training protocols. However, these studies were not performed with trained athletes, which may partly explain the conflicting findings in our study.

A limitation of the present study is that two interventions are compared without a classical control group, since we wanted to match the training time in the two interventions. Moreover, it was not possible to monitor physical and physiological responses during the entire training period to describe the full external and internal loading, since the players were assessed during a real setting pre-season period.

## Conclusions

In conclusion, added high-intensity intermittent exercise training, organised as speed endurance training drills, improves skeletal muscle oxidative capacity and exercise performance to a greater degree than added moderate intensity training, organised as small-sided soccer games, in well-trained male soccer players, with similar group responses in muscle lactate^−^/H^+^ cotransporter and antioxidative capacity. In contrast, moderate intensity training increased muscle GLUT4 expression and tended to induce greater upregulation in Na^+^–K^+^ ATPase subunit expression and muscle glycogen storage capacity than high-intensity interval training.

## Abbreviations

AAT: Arrowhead agility test
ANOVA: Analysis of variance
ATP: Adenosine triphosphate
BSA: Bovine serum albumin
CAT: Catalase
CS: Citrate synthase
CV: Coefficient of variation
DTT: Dithiothreitol
EDTA: Ethylenediaminetetraacetic acid
EGTA: Ethylene glycol tetraacetic acid
ES: Effect size
FXYD1: Phospholemman protein
GLUT-4: Glucose transporter type 4
GPX: Glutathione peroxidase
HAD: Beta-hydroxyacyl-CoA-dehydrogenase
HCl: Hydrogen chloride
HEPES: 4-(2-Hydroxyethyl)-1-piperazineethanesulfonic acid
HIR: High-intensity running distance (*m* > 14 km/h)
HRP: Horseradish peroxidase
HSR: High-speed running distance (*m* > 21 km/h)
Ia: Intense accelerations (*n* > 3 m/s^2^)
Id: Intense decelerations (*n* > 3 m/s^2^)
MCT4: Monocarboxylate transporter 4
NaCl: Sodium chloride
Na3VO4: Sodium orthovanadate
NF-κΒ: Nuclear factor kappa-light-chain-enhancer of activated B cells
NHE1: Na^+^/H^+^ exchanger isoform 1
NP-40: Tergitol-type NP-40 (nonylphenoxypolyethoxylethanol)
PGC-1α mRNA: Peroxisome proliferator-activated receptor-γ coactivator messenger ribonucleic acid
PFK: Phosphofructokinase
PVDF: Polyvinylidene difluoride
ROS: Reactive oxygen species
RST: Repeated sprint test
SDS: Sodium dodecyl sulphate
SET: Speed endurance training
SOD1, SOD2: Superoxide dismutase 1 and 2
SSG: Small-sided games
TBST: Tris-buffered saline including Tween-20
TCA cycle: Tricarboxylic acid cycle
TD: Total distance
Yo–Yo IR2: Yo–Yo Intermittent Recovery test level 2
